# New Concepts in Molecular Imaging: Non-Invasive MRI Spotting of Proteolysis Using an Overhauser Effect Switch

**DOI:** 10.1371/journal.pone.0005244

**Published:** 2009-04-27

**Authors:** Philippe Mellet, Philippe Massot, Guillaume Madelin, Sylvain R. A. Marque, Etienne Harte, Jean-Michel Franconi, Eric Thiaudière

**Affiliations:** 1 INSERM IFR 004, and Magnetic Resonance Center CNRS UMR 5536 University Victor Segalen Bordeaux, Bordeaux, France; 2 Magnetic Resonance Center CNRS UMR 5536 University Victor Segalen Bordeaux, Bordeaux, France; 3 Université de Provence CNRS UMR 6264 Case 521, Marseille, France; 4 CNRS, UPR 8641, Centre de Recherche Paul Pascal (CRPP), Université de Bordeaux, Pessac, France; University of Arkansas for Medical Sciences, United States of America

## Abstract

**Background:**

Proteolysis, involved in many processes in living organisms, is tightly regulated in space and time under physiological conditions. However deregulation can occur with local persistent proteolytic activities, e.g. in inflammation, cystic fibrosis, tumors, or pancreatitis. Furthermore, little is known about the role of many proteases, hence there is a need of new imaging methods to visualize specifically normal or disease-related proteolysis in intact bodies.

**Methodology/Principal Findings:**

In this paper, a new concept for non invasive proteolysis imaging is proposed. Overhauser-enhanced Magnetic Resonance Imaging (OMRI) at 0.2 Tesla was used to monitor the enzymatic hydrolysis of a nitroxide-labeled protein. *In vitro*, image intensity switched from 1 to 25 upon proteolysis due to the associated decrease in the motional correlation time of the substrate. The OMRI experimental device used in this study is consistent with protease imaging in mice at 0.2 T without significant heating. Simulations show that this enzymatic-driven OMRI signal switch can be obtained at lower frequencies suitable for larger animals or humans.

**Conclusions/Significance:**

The method is highly sensitive and makes possible proteolysis imaging in three dimensions with a good spatial resolution. Any protease could be targeted specifically through the use of taylor-made cleavable macromolecules. At short term OMRI of proteolysis may be applied to basic research as well as to evaluate therapeutic treatments in small animal models of experimental diseases.

## Introduction

Proteases are enzymes which catalyze the hydrolysis of the peptide bonds in proteins. The selectivity for a specific amino acid sequence may be broad or narrow depending on the protease involved. Proteases are present in every extra- and intra-cellular compartment [1]. Since uncontrolled proteolysis can be very deleterious for tissues, proteases (other than those found in the digestive track) are stored as inactive pro-enzymes that are activated only for specific and localized tasks limited in time. A tight control of the proteolytic activity is also performed by numerous endogenous protease inhibitors present at high concentration in all tissues or by compartmentalization like in the lysosomes.

The Human Genome Project revealed more than 500 protease-encoding genes [2]. The substrate specificity, the trigger events and site of activation are still unknown for most of them. However, it is known that transient proteolysis is involved in many physiological situations like inflammation, coagulation, fibrinolysis, hormone generation, development or tissue turnover. Interestingly, persistent proteolysis has been observed in many diseases like cystic fibrosis, emphysema, rheumatoid arthritis, bacterial, viral and parasitic infections, tumor and metastasis spreading or pancreatitis. In the intracellular compartment apoptosis also involves specific proteolytic cascades.

Thus many physiological and pathological events are closely related to persistent protease activity. From the point-of-view of integrative biology, the question arises whether those events could be detected and followed in-vivo in an intact organism by revealing the corresponding proteolytic activity. Non invasive imaging methods using protease-specific contrasts agents are natural candidates for this purpose. Furthermore detecting an enzymatic activity offers the possibility of signal amplification via a renewable substrate.

The first evidence that protease imaging is a pertinent way to study diseases in vivo was given using near infrared fluorimetry on a murine tumor model. Self quenched fluorescent peptides were actually cleaved and were able to generate a detectable signal in the tumor environment [3], [4]. In spite of recent progresses optical methods have strong limitations due to the light transmission to and from deep-seated organs. With this respect Magnetic Resonance Imaging (MRI) constitutes a good alternative. Interesting protease and glycosidase substrates acting as MRI contrast agents have been proposed [5]–[11]. However lower toxicity and much higher contrasts are needed to compensate for the low sensitivity of nuclear magnetic resonance. Overhauser Magnetic Resonance Imaging (OMRI) has the potential to significantly enhance the sensitivity of MRI [12]–[19]. It is a double resonance experiment that transfers a fraction of the higher magnetization of the electron of a free radical to the protons of surrounding water molecules. Recently OMRI was successfully applied to in vivo oxymetry imaging by correlating the Electron Paramagnetic Resonance (EPR) line width variation of a trityl free radical to oxygen concentration [20].

Nitroxides are a family of stable free radicals. Several biocompatible nitroxides have been used in EPR [21] and OMRI [15] experiments *in vivo*. The Overhauser enhancement strictly depends on the nitroxide EPR line width. Due to the nitroxide asymmetric structure their EPR spectra significantly widen and flatten as their rotational correlation times increase [22]. Here this property was applied to design a contrast agent sensitive to proteolysis and detectable through Overhauser enhancement.

In this paper a general molecular imaging method with generation of high positive contrast in the presence of proteolytic activity is proposed. As an example nitroxides were covalently bound to bovine serum albumin (BSA). An experimental setup for OMRI at 0.2 T was built so that dynamic nuclear polarization can occur at depth in the range of a centimeter without any significant heating of the sample. OMRI of the nitroxide-labeled BSA sample at 0.2 T revealed no signal enhancement because of the elevation of the nitroxide correlation time. The initial magnetic resonance image intensity was strongly enhanced by enzymatic digestion of the carrier protein. Such a protease-switch method can be adapted to any proteolytic activity by linking a nitroxide to a large carrier molecule through specifically cleavable peptide substrates.

## Results

### Sensitivity

**Figure 1:**
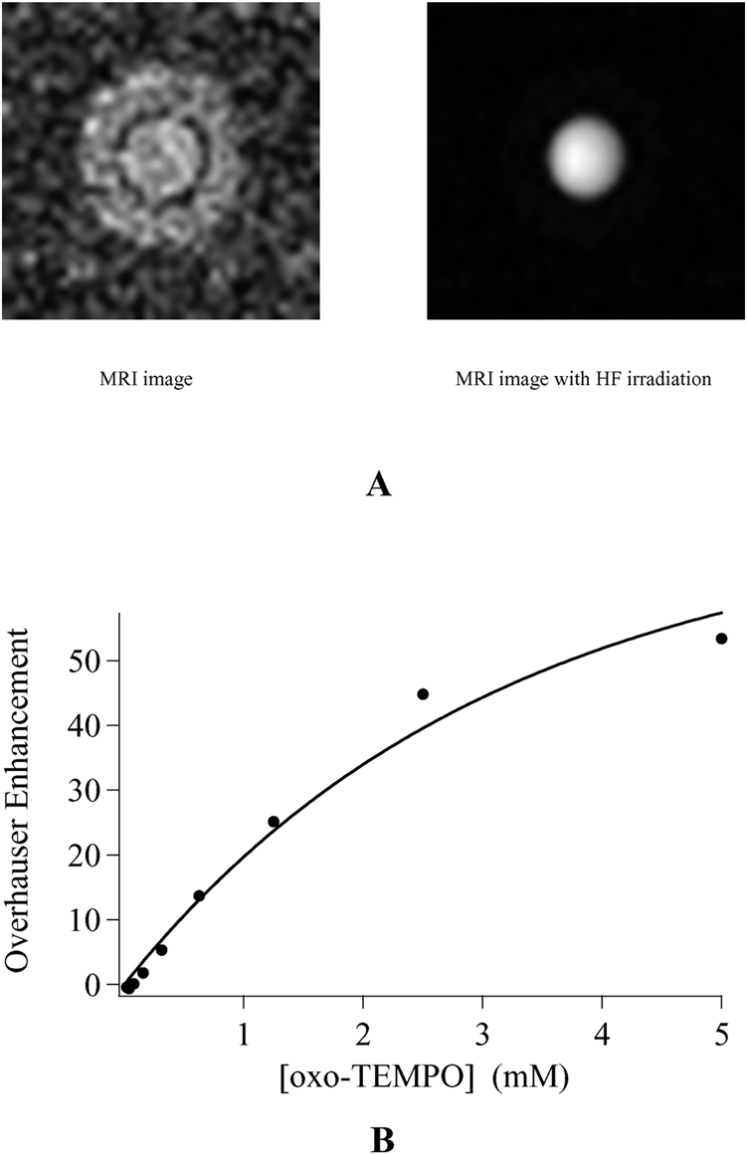
Sensitivity of 2D OMRI probed by successive dilutions of a 5 mM solution of Oxo-TEMPO. (A) MR images of a 5 mm tube of 1 mM Oxo-TEMPO in water inserted into a 10 mm tube filled with pure water. Left: without HF irradiation (window level = 12, window width = 24); Right: with HF irradiation (window level = 214, window width = 428). The imaging parameters were: Field of View 25×25 mm^2^, matrix 64x64, slice thickness 5 mm, TR = 800 ms, TE = 28 ms, saturation time 670 ms. (B) Overhauser enhancement versus Oxo-TEMPO concentration. The continuous line is the curve fit to Eq. A4 (Text S1), with s = 0.7.

Figure 1A highlights the sensitivity of OMRI at 0.2 T on a phantom containing 1 mM 4-Oxo-2,2,6,6-tetramethyl-1-piperidinyloxy free radical (Oxo-TEMPO) in aqueous solution. Using 670 ms hyperfrequency (HF) irradiation and a recovery time (TR) of 800 ms a clear signal enhancement is visible. The concentration dependence of the OMRI setup was probed by imaging successive dilutions of an Oxo-TEMPO solution, after N_2_ bubbling to remove dissolved O_2_. For each concentration two 2D images were recorded, one without and one with HF irradiation. The observed signal enhancement versus Oxo-TEMPO concentration plot is given in Figure 1B. The Overhauser effect generates a contrast for concentrations as low as 0.2 mM of Oxo-TEMPO. At 5 mM signal amplification reaches 54 times the intensity level of the initial MRI image. More significantly an enhancement of 25 remains at 1.25 mM Oxo-TEMPO which is a concentration compatible with future *in vivo* experiments. It was necessary to reach high enhancements *in vitro* because T_1_ values are such that the leakage factor f = 1−(T_1_/T_10_) (see Text S1) might be much lower than 1 in vivo. From the relaxivity r_1_ of Oxo-TEMPO (0.5 s^−1^mM^−1^) and the T_10_ of pure water (3.46 s), it was possible to calculate the leakage factor throughout the concentration range of Figure 1B (see Eq. A3 in Text S1). Assuming an electron-nucleus coupling constant ρ = ½, curve fitting of the data to Eq. A4 (Text S1) gave a saturation factor of 70%. Such incomplete saturation might be due to power loss in the HF transmission line. It is of note that the combined effects of the gated mode HF irradiation and the design of the cavity for a nearly pure magnetic mode actually prevented any significant heating of the samples.

### 3D experiments

**Figure 2:**
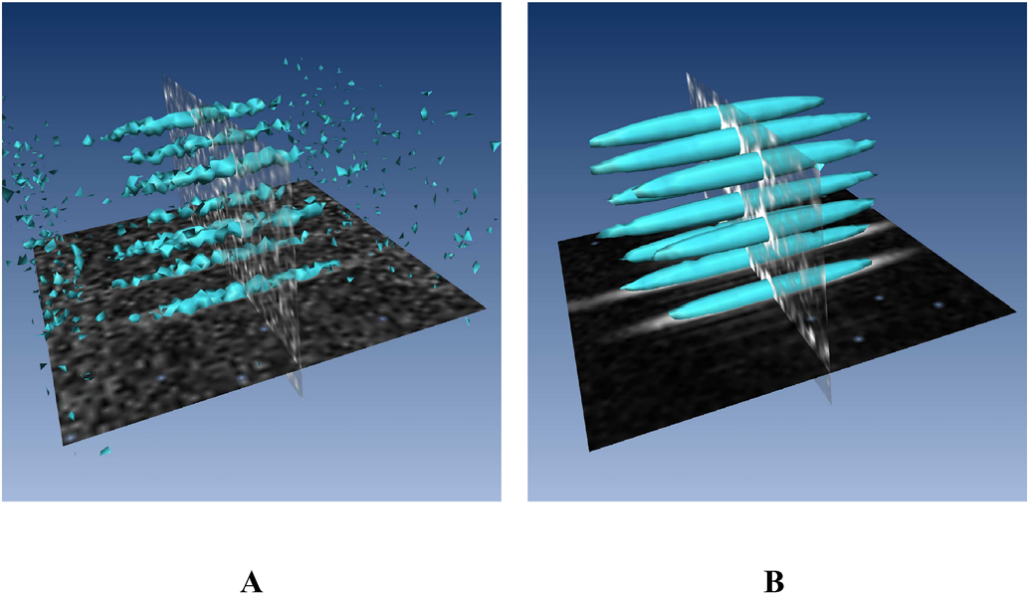
Isosurface representation of 3D OMRI of nine capillaries filled with 5 mM Oxo-TEMPO. (A) without HF irradiation (threshold = 42); (B) with HF irradiation (threshold = 400).

Future *in vivo* applications of proteolysis imaging will require an accurate localization of the signal enhancement by the nitroxides in three dimensions. The experimental setup was challenged with a phantom made of nine capillaries of 1.4 mm internal diameter filled with 5 mM Oxo-TEMPO inside an empty 20 mm diameter tube. Figure 2 shows that each capillary is clearly individually delimited as expected with an isotropic resolution of 0.5 mm in 3D. Such a resolution obtained at a static field of 0.2 T is unusual and is compatible with mouse imaging. Furthermore a signal enhancement of around 40 upon HF irradiation has been measured throughout the whole field of view.

### Proteolysis of nitroxide-labelled BSA

**Figure 3:**
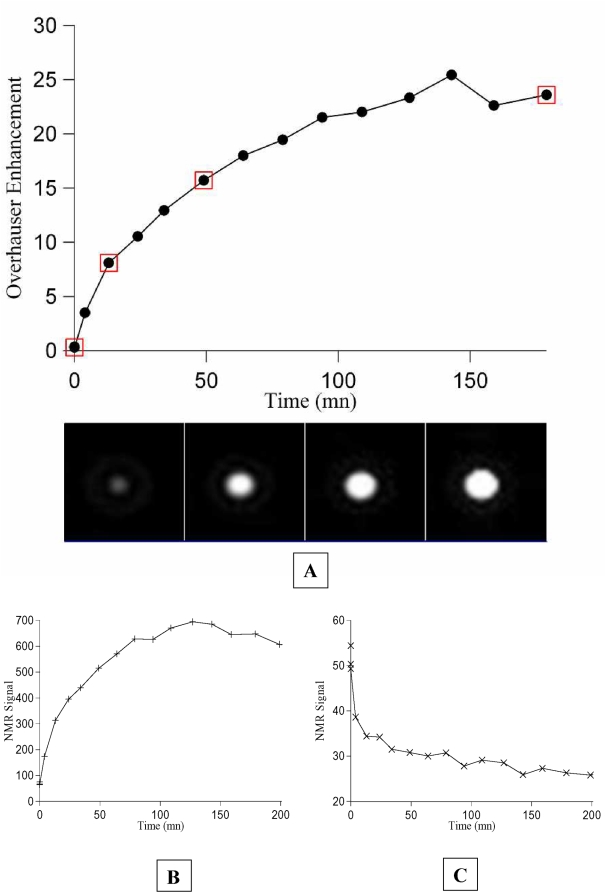
2D OMRI monitoring of the proteolytic digestion of nitroxide-labeled BSA by papain. (A) Overhauser enhancement versus time. The images corresponding to the points highlighted by empty squares are displayed underneath in the same order. (B) NMR signal in the presence of HF irradiation versus time. (C) NMR signal in the absence of HF irradiation versus time.

**Figure 4:**
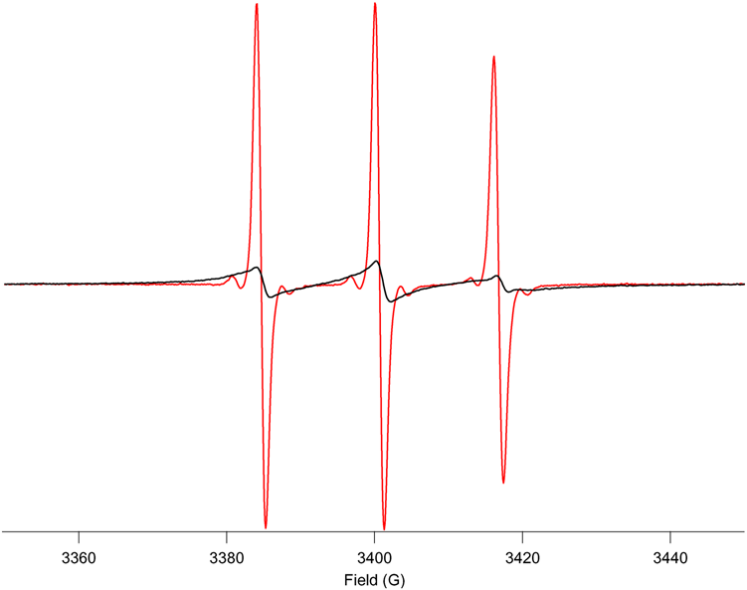
X Band ESR spectra of the nitroxide-labeled BSA solution before (red line) and after (blue line) proteolytic digestion. The spectra have been normalized to the same peak area.

A solution of nitroxide-labeled BSA with an estimated 2 mM content of bound nitroxide was probed with the OMRI setup. No significant signal enhancement was recorded in the presence of HF irradiation (see Figure 3). A high concentration of the proteinase papain was then added and images without and with HF irradiation were recorded as a function of time. Figure 3A shows that the signal enhancement increases with time until it reaches a plateau after about 140 minutes. Figure 3B and 3C show that the final Overhauser enhancement of 24 is due to both a high increase of the signal with HF irradiation and also partly to a slight decrease of the signal without HF irradiation because of change in the T_1_ upon digestion. Indeed the longitudinal relaxation times T_1_ before and after proteolysis were measured. It shifted from 390 ms to 1300 ms upon digestion by papain. With a recovery time of 500 ms, Ernst equations predict a steady state equilibrium magnetization ratio M_peptides_/M_BSA_ = 0.45. The experimental image intensity ratio between the digested peptides and BSA is 0.44 (see Figure 3). Thus the variation of T_1_ entirely explains the slight decrease of the image intensity without HF irradiation upon digestion of the sample. The T_1_ variation itself is easily explained by the variation of the correlation time of the nitroxide-carrying species. The τ_c_ value is about 42 ns on the intact BSA[23] which is close to the theoretical T_1_ lower point at τ_c_ = 1/ω_0_  =  19.3 ns at 0.2 Tesla. After digestion, τ_c_ can be estimated from the EPR spectrum to 76 ps (see Figure 4 and Equation 1 below). This T_1_-shift upon digestion predicts a lesser efficiency of relaxation by surrounding paramagnetic species. Noteworthy, the assumed value for the concentration in the nitroxide radical is compatible with both the Overhauser enhancement and the T_1_ values measured at the end of the digestion. From Figure 1 the enhancement final value of 24 obtained after proteolysis suggests an Oxo-TEMPO concentration of 1.3 mM, to be compared to the expected concentration of 2 mM. This apparent discrepancy can be explained by the presence of O_2_ in the proteolysis experiments, where the samples were left with their spontaneous content of dissolved O_2_ like *in vivo* conditions. Other experiments (not shown) suggested that degassing brought an additional signal enhancement of about 60%, in agreement with an expected nitroxide concentration of 2 mM. Similarly, a T_1_ value of 1300 ms was observed after digestion, which corresponds to a nitroxide concentration of 1.7 mM from relaxivity data at 0.2 T (not shown). Considering that the digestion by papain is unlikely to be complete [1] the results are in agreement with the initial assumption of 2 mM content in nitroxide and thus with a stoichiometric reaction of the succinimidyl ester derivative of the nitroxide on BSA with an average of 30 radicals per BSA molecules.

The X-band EPR spectra measured before and after proteolytic digestion of the labelled BSA provide a clear explanation of the increase in Overhauser enhancement (Figure 4). Before proteolysis the EPR spectrum is notably broadened when compared to a free nitroxide molecule. This is typical of a spectrum obtained in the slow tumbling regime, with a strong reduction of the spin-spin relaxation rate constant of the electron spin bound to a protein with a correlation time τ_c_ = 42 ns [23] at 5.4 GHz EPR frequency [22]. From Eq. A5 (Text S1) it can be anticipated that this decrease in T_2S_ likely leads to the cancellation of the saturation factor s. As described previously [20] OMRI is very sensitive to the EPR line width and here the Overhauser effect is completely suppressed before proteolytic digestion. Figure 4 confirms that after proteolytic digestion the nitroxide returns to the fast rotational regime compatible with the Overhauser effect. To quantify the return to near fast isotropic motion the following equation was used to estimate the final correlation time τ_c_ [24]:
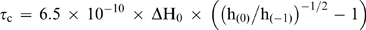
(1)where ΔH_0_ is the peak to peak line width of the central line in gauss and h_(0)_ and h_(−1)_ are the peak to peak amplitudes of the central and high field lines, respectively. Equation 1 applied to the spectrum of the digested sample yields τ_c_ = 76 ps, a value which suggests the production of free nitroxides or short labeled peptides. BSA is a highly structured protein made of helices and short loops. Thus papain is likely unable to carry out complete digestion up to the amino-acid level. Rather the digestion yielded short peptides, in agreement with the calculated correlation time of 76 ps.

Thus the recovery of Overhauser enhancement is accounted for by the protease digestion of the high molecular weight protein into low molecular weight peptides. This system is an effective protease-activated switch for Overhauser-enhanced MRI.

## Discussion

In this paper a non invasive method designed to perform MRI of the proteolytic activity in deep-seated organs is described. This method needs a magnetic resonance imaging system including a resonant cavity tuned on the free electron EPR frequency of a nitroxide. With a very simple biochemical model, it is demonstrated that a proteolytic enzyme can modulate the Overhauser effect through the alteration of the motional correlation time of a nitroxide labelled substrate. The detection of proteolysis is achieved by recording two proton magnetic resonance images, one in the absence and one in the presence of HF irradiation. In the presence of a free nitroxide the Overhauser enhancement can exceed 50. Before proteolysis the nitroxide molecules are bound to the high molecular weight substrate associated with a slow tumbling regime which alters their EPR spectrum enough to suppress the Overhauser effect. Proteolysis regenerates a fast motional regime for the nitroxide molecules and hence the Overhauser effect. Here the MRI signal is 25 fold higher upon proteolysis of the substrate. This signal amplitude is a considerable progress compared to the proteolysis imaging methods proposed previously [5]–[11]. Image interpretation is simple: any significant increase in signal indicates proteolysis. An easy way to read out the Overhauser effect is to calculate enhancement images instead of magnitude images.

The EPR cavity has a practical diameter of 2.5 cm which easily accommodates a living mouse. Furthermore the microwave field at 5.4 GHz reaches the center of a 2 cm diameter tube filled with nitroxide in water (not shown). Importantly, sample heating is avoided. Thus, by linking a nitroxide to a carrier molecule through peptides specific for any chosen proteinase target, numerous in vivo applications are possible at short term: basic research on proteolysis and physiological events including developmental biology that involve intense tissue remodeling; non-invasive studies of newly identified proteinase activities; pharmaceutical research, particularly to monitor in longitudinal studies proteolytic activity during an experimental anti-proteinase treatment without the need to sacrifice the animals.

Future developments may need even more sensitive detection of the proteolytic activity. The Overhauser effect can be improved by building substrates with ^15^N labelled nitroxides. The ½ spin of ^15^N reduces the number of EPR lines to 2 thus enhancing each line intensity. Moreover the line width of the nitroxides can be reduced by synthesizing molecules deuterated at the sites coupled to the free electron.

**Figure 5:**
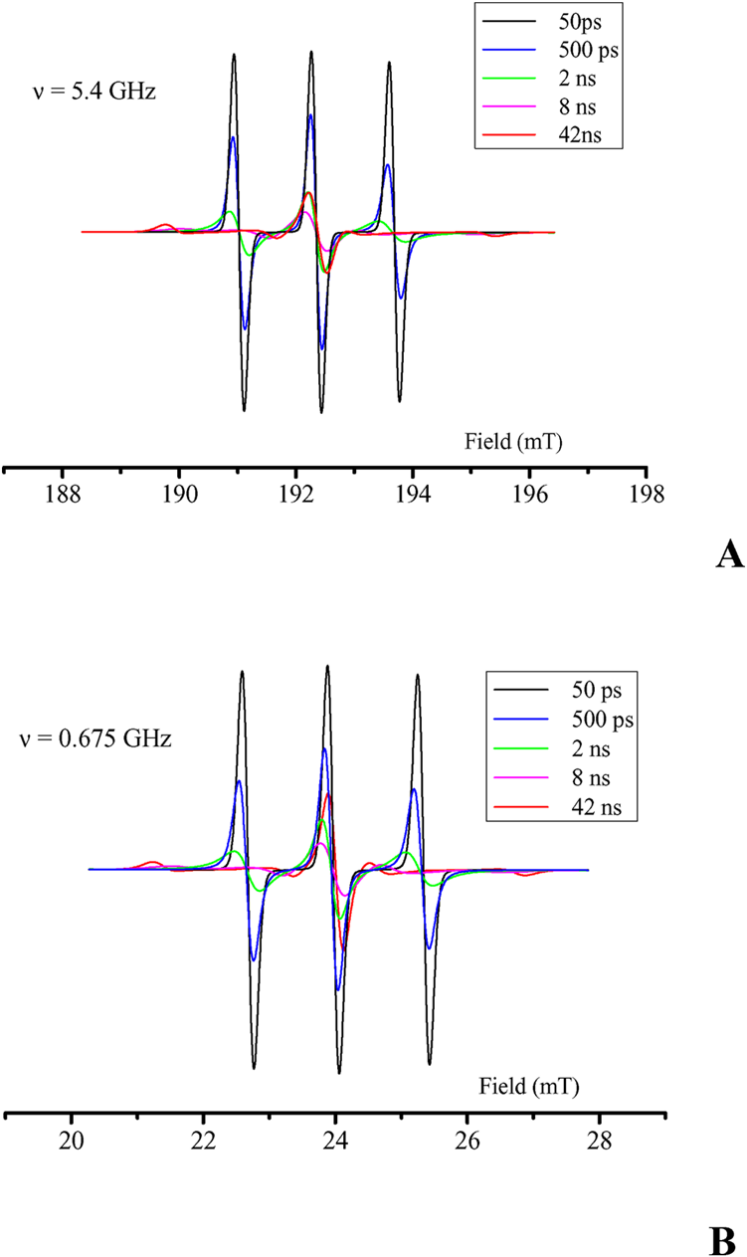
Simulated ESR spectra of a typical nitroxide for a range of motional correlation times τ_c_. (A) Spectra calculated with B_0_ = 0.2 Tesla (ν = 5.4 GHz). (B) Spectra calculated with B_0_ = 25 mTesla (ν = 0.675 GHz).

Long term applications to larger animals or humans will require some changes. The main concern is the HF irradiation frequency. To enhance the penetration of HF into deeper-seated tissues the frequency must be lowered and hence the B_0_ field. The simulations presented in Figure 5 show that with B_0_ field divided by 8 (25 mT) there is still a strong effect of the motional correlation time on the EPR spectra on the central line and even a stronger effect on the low-field and high-field lines of the nitroxide. Slow tumbling conditions prevail at a field as low as 25 mTesla (corresponding to an EPR frequency of 675 MHz) for a carrier with a molecular weight of 4kDa only. The effect is also present at intermediate B_0_ field values (not shown). Thus the same substrate designs and imaging principles can be used at low field on larger animals. This would open the way to new diagnostic methods for tumors and metastasis since proteolysis in the surrounding tissues is an early event in these pathologies[25]. Interestingly, the intensity of the proteolysis activity is most probably independent on the size of the tumor and could reveal some events undetected by other imaging methods. This would be particularly beneficial since, despite constant progress in the therapies against malignancy, survival rates to a cancer is still strongly correlated to the precocity of the diagnosis [26].

## Materials and Methods

### Magnetic Resonance Imaging equipment

MRI experiments were performed on a whole body open system OPEN-Viva (Siemens AG Germany), with the non-magnetic EPR cavity placed at the center of the 40cm magnet bore. Dynamic Nuclear Polarization was carried out at a fixed magnetic field B_0_ of 0.193 T, resulting in proton magnetic resonance frequency of 8.24 MHz and a EPR resonance frequency of 5.435 GHz for the central line of the nitroxide radical Oxo-TEMPO. The B_0_ field was vertically oriented along the Y gradient axis.

A home-made transmit-receive saddle-shaped coil of 26 mm inner diameter and length was inserted in the cavity, with the manual tuning circuitry placed apart. The radio-frequency (RF) field of this magnetic resonance (MR) coil was aligned with the Z axis of the MRI scanner. A transmit/receive switch device was inserted in the RF control unit of the MRI equipment.

**Figure 6:**
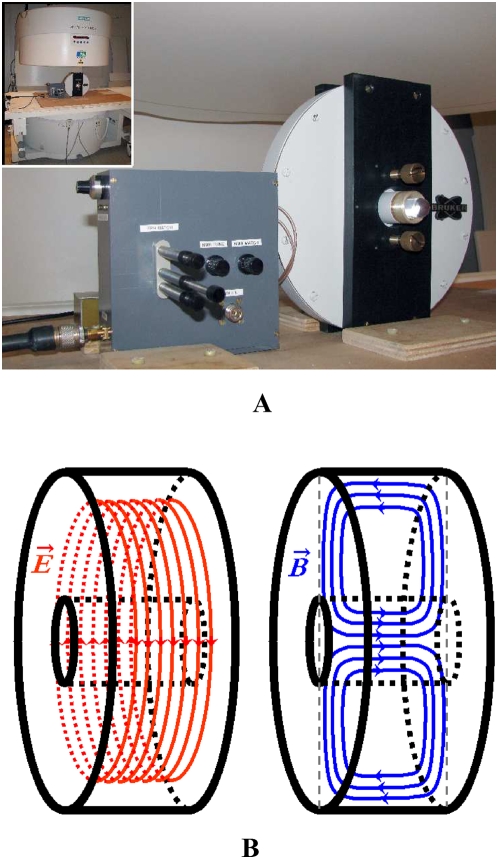
The OMRI experimental setup. (A) the EPR cavity in the 0.2 T imaging system. (B) Simulation of the electric field E (red) and the magnetic field B (blue) in the resonant EPR cavity in TE011 mode. The electric field is concentrated outside the sample housing (black inner cylinder) while the magnetic field is centered on the sample.

The EPR cavity (Figure 6) consisted in a non-magnetic cylindrical cavity operating in TE011 mode made by Bruker (Wissembourg, France). Importantly, its geometry was designed to minimize the electric component of the electromagnetic hyperfrequency (HF) field in the center of the cavity, thus minimizing sample warming upon microwave emission. The HF magnetic field was maximal in the center of the cavity where the sample was placed, and aligned with the X axis of the MRI scanner. The HF field was generated by a synthesizer and amplified with a cascade of two specific amplifiers (RFPA, Artigues-pres-Bordeaux, France) then fed with a coaxial cable to a wave guide, including matching and tuning devices, connected to the cavity. The cavity was tuned and matched in the presence of the sample with the help of a network analyzer.

The size of the cavity has been matched to the resonance frequency of the central line of a nitroxide EPR spectrum such as Oxo-TEMPO with an adjustable range of ±50 MHz.

The emission of microwaves was synchronized with a programmable external pulse generator (RFPA, France), triggering both the HF generator and the MR acquisition in a repeatable cycle.

### Pulse sequences

Two-dimension (2D) magnetic resonance images were obtained with a sequence derived from a standard spin echo, using a non-selective 180° refocusing pulse to minimize eddy-currents that could take place in the end plates of the cavity due to the slice selection gradient pulse. The HF pulse width was in the range of 400 ms to 700 ms compatible with the T_1_ value of water in the presence of nitroxide in the millimolar range, followed by the MRI sequence with an echi time (TE) of 28 ms and minimal TR of 78 ms.

All MR adjustments were done manually, using the same fixed receiver gain for both measurements, without and with HF irradiation, so that signals can be directly compared and Overhauser enhancement calculated.

For 3D imaging of capillaries containing free radicals a Gradient-Echo sequence with a non-selective excitation pulse was used, with a TE of 16 ms and a minimal TR of 54 ms. The effective TR with HF irradiation was 140 ms, resulting from a 80 ms HF pulse followed by 60 ms for NMR acquisition. The geometrical parameters were: Field of View  = 16*28*28 mm3 (Slice*Read*Phase, respectively) and matrix 32*64*64 resulting in a 0.5*0.44*0.44 mm3 resolution. The total acquisition time was 4′08″. Due to limitations in the efficiency of the HF transmission line, the HF irradiation was applied only for the central lines of the k-space, corresponding to half of the total number of echo acquisitions.

### Overhauser enhancement

Overhauser enhancement (E) was calculated as:
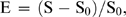
where S and S_0_ are the NMR signals measured with and without HF irradiation, respectively.

### T_1_ measurements

Longitudinal relaxation time T1 measurements of all samples were measured using an inversion-recovery technique with a single-shot, line-reordered gradient echo sequence. The magnetization was first inverted by a 180° RF pulse, followed by an inversion delay TI before MRI acquisition. The signal of this image is proportional to the longitudinal magnetization at the time TI after the inversion pulse. TI was systematically varied and the T_1_ value has been extracted from signal curve evaluation with post-processing software Igor Pro (Wavemetrics, Lake Oswego, OR).

### Nitroxide-labeled BSA

Nitroxide free radicals were covalently bound to the BSA carrier molecule through the lysine residues. 145 mg of BSA (fraction V, Sigma) were dissolved in 1 ml of 50mM HEPES buffer, 0.15 M NaCl at pH 8.3. A thirty times molar excess (17 mg) of the amine reactive nitroxide 1-Oxyl-2,2,5,5-tetramethylpyrroline-3-carboxylate N-Hydroxysuccinimide Ester (Toronto Research Chemicals Inc.,Canada) was dissolved in 200 μl of DMSO. The nitroxide solution was slowly added to the BSA solution during one hour under stirring at constant pH = 8.3 at room temperature and the reaction was let another hour. Labelled BSA was then purified on a PD10 desalting column (GE Healthcare) loaded with 50 mM HEPES, 0.15 M NaCl at pH 7.4. A 30/1 labelling ratio corresponding to 100% labelling yield was assumed for further experiments.

### Enzymatic digestion of nitroxide-labeled BSA

The nitroxyde-labeled BSA solution was diluted to obtain about 2 mM nitroxide concentration. The sample (2.4 ml) was transferred into a 5-mm diameter quartz tube positioned at the center of a 10 mm quartz tube filled with water. This setting was necessary to optimize the filling factor of the NMR coil. The proteolytic digestion was initiated by adding 100 μl of a solution of 10 mg/ml papain (lyophilized powder from Papaya Latex, Sigma) and 20 mM Cysteine (Aldrich) as papain activator. 2D MRI and OMRI images were then continuously recorded as described above.

### EPR spectra

EPR experiments were made with an ESP300E Bruker (France) X band spectrometer operating at a microwave frequency of 9.3 GHz.

### EPR spectra simulations

EPR spectra simulations were performed with the Easyspin Program (version 3.0.0)[27] supported on Matlab software (version 7.1.0), using the chili simulation function with the conventional Landé factor *g* and nitrogen hyperfine coupling constant *a*_N_ for nitroxides such as TEMPO, namely gxx = 2.008, gyy = 2.006, gzz = 2.003 (g = 2.0063) and Axx = 5G, Ayy = 5G, Azz = 30G (A = 13.3G). Simulated spectra were calculated at frequencies of 5.4 and 0.675 GHz for correlation times τ_c_ of 50 ps, 500 ps, 2 ns, 8 ns and 42 ns. Those τ_c_ values correspond to molecular weights of the nitroxide-carrying peptide/protein of 0 (free nitroxide), 1, 4, 16 and 66 kD, respectively.

## Supporting Information

Text S1 Theoretical Background of Dynamic Nuclear Polarization
(0.04 MB DOC)
