# Supplementary material for: New Concepts in Molecular Imaging: Non-Invasive MRI Spotting of Proteolysis Using an Overhauser Effect Switch
Source: PLoS One. 2009 Apr 27;4(4):e5244. doi: 10.1371/journal.pone.0005244 (PMC2671144; doi:10.1371/journal.pone.0005244)
Supplement: Text S1 — Theoretical Background of Dynamic Nuclear Polarization (0.04 MB DOC) [file pone.0005244.s001.doc]

**Supporting Information**

Theoretical Background of Dynamic Nuclear Polarization

The general theory of Dynamic Nuclear Polarization is well described elsewhere[1,2]. Here only the specific case of the interaction between an electron spin ***S*** carried by a radical species and a proton spin ***I*** in liquids in a constant magnetic field B_0_ shall be considered. More details can be found in the litterature[3-10].

The Overhauser effect (E) on spin ***I*** that occurs upon EPR irradiation (usually by microwaves) of spin ***S*** coupled to it can be calculated as:

$E=\frac{I_{Z}}{I_{0}}-1=\rho.f\frac{\left(S_{0}-S_{Z}\right)}{I_{0}}$ (A1)

where I_Z_ and I_0_ are the observed and equilibrium polarization of the nuclear spin, S_Z_ and S_0_ the observed and equilibrium polarization of the electron spin, **ρ** the electron-nucleus coupling constant and **f** the leakage factor.

Assuming a pure dipolar interaction under the extreme narrowing limit, the electron-nucleus coupling constant ρ can be approximated to ½. The leakage factor **f** represents the part of the electron-nucleus interaction in the longitudinal relaxation of the nucleus:

$f=1-\frac{T_{1I}}{T_{1I0}}$ (A2)

where T_1_*_I_* and T_1_*_I_*_0_ are the T_1_ values of the nucleus observed in the presence and the absence of the radical species. Knowing the spin-lattice relaxivity r_1_ and the concentration c of the latter, **f** can be written as:

$f=\frac{r_{1}.c.T_{1I0}}{\left(1+r_{1}.c.T_{1I0}\right)}$ (A3)

The saturation factor **s** of spin ***S*** induced by EPR irradiation can be introduced in the formulation of the Overhauser effect E as:

$E=\frac{\rho.f.s.\gamma_{S}}{\gamma_{I}}$ (A4)

where γ*_S_* and γ*_I_* are the magnetogyric ratio of the corresponding spins. The saturation factor is described as:

$s=\frac{\gamma_{S}^{2}.B_{1}^{2}.T_{1S}^{2}.T_{2S}^{2}}{1+\gamma_{S}^{2}.B_{1}^{2}.T_{1S}^{2}.T_{2S}^{2}+\left(\omega-\omega_{S}\right)^{2}.T_{2S}^{2}}$ (A5)

where T_1_*_S_* and T_2_*_S_* are the spin-lattice and the spin-spin relaxation time constants of spin ***S***, B_1_ the irradiating magnetic field oscillating at angular frequency ω and ω_S_ the resonance frequency of the electron. The value of **s** ranges from zero in case of no saturation to unity in case of complete saturation.

In case of an additional hyperfine coupling of the electron with an adjacent nucleus ***K***, a number of possible transitions between spin states can occur hence resonance splitting of the EPR lines. In nitroxides the electron is coupled with a hyperfine constant A_SK_ to the ^14^N nucleus (K=1). Six spin states can then be defined and 6 possible transitions. At “high fields” ($B_{0}>>2\pi.\frac{A_{KS}}{\gamma_{S}}$), only 3 transitions between spin states are considered, with 3 resonances in the nitroxide EPR spectrum. As only one EPR line can be saturated, the Overhauser effect is divided by the multiplicity n (n=3).

Taking into account the saturation time TS of a given EPR line the Overhauser effect can be written as:

$E=\left(\frac{\rho.f.s}{n}\right).\left(\frac{\gamma_{S}}{\gamma_{I}}\right).e^{-\left(\frac{T_{S}}{T_{1I}}\right)}$ (A6)

Under the experimental conditions of the present study, the maximal value for E, *i.e.* the maximal possible enhancement in the MRI signal, is around 110.

1. Overhauser AW (1953) Polarization of nuclei in metals. Phys Rev: 411-412.

2. Abragam A, Combrisson J, Solomon I (1957) Compt Rend Acad Sci Paris: 157.

3. Abragam A (1978) Principles of Dynamic Nuclear Polarization. Rep Prog Phys 41: 395-467.

4. Hausser KH, Stehlik D (1968) Dynamic Nuclear Polarization in Liquids. Adv Magn Reson 3: 79-139.

5. Dwek RA, Richards RE, Taylor D (1969) Nuclear Electron Double Resonance in Liquids. Ann Rev NMR Spectr 2: 293-344.

6. Guiberteau T, Grucker D (1993) Dynamic Nuclear Polarization of Water Protons by Saturation of s and p EPR Transitions of Nitroxides. J Magn Reson A 105: 98-103.

7. Guiberteau T (1994) Polarisation dynamique en champ magnétique faible : Application à la spectroscopie et à l’oxymétrie. Strasbourg, France: Université Louis Pasteur.

8. Grucker D, Guiberteau T, Eclancher B, Chambron J, Chiarelli R, et al. (1995) Dynamic Nuclear Polarization with Nitroxides Dissolved in Biological Fluids. J Magn Reson B 106: 101-109.

9. Guiberteau T, Grucker D (1996) EPR Spectroscopy by Dynamic Nuclear Polarization in Low Magnetic Field. J Magn Reson B 110: 47-54.

10. Ardenkjaer-Larsen JH, Laursen I, Leunbach I, Ehnholm G, Wistrand LG, et al. (1998) EPR and DNP properties of certain novel single electron contrast agents intended for oximetric imaging. J Magn Reson 133: 1-12.
